# The immunomodulating activity of trimodulin (polyvalent IgM, IgA, IgG solution): a post hoc analysis of the phase II CIGMA trial

**DOI:** 10.1186/s13054-023-04719-9

**Published:** 2023-11-09

**Authors:** Mervyn Singer, Antoni Torres, Corina C. Heinz, Sabrina Weißmüller, Alexander Staus, Steffen Kistner, Ksenia Jakubczyk, Thomas Häder, Patrick Langohr, Andrea Wartenberg-Demand, Jörg Schüttrumpf, Jean-Louis Vincent, Tobias Welte

**Affiliations:** 1 Division of Medicine, Bloomsbury Institute of Intensive Care Medicine, University College London, London, UK; 2 Hospital Clínic, Servei de Pneumologia I Allèrgia Respiratòria, Catedràtic de Medicina, Universitat de Barcelona, Barcelona, Spain; 3 IDIBAPS, ICREA, CIBER de Enfermedades Respiratorias, Barcelona, Spain; 4 Biotest AG, Landsteinerstraße 5, 63303 Dreieich, Germany; 5 Department of Intensive Care, Erasme University Hospital, Brussels, Belgium; 6 Klinik für Pneumologie, Medizinische Hochschule Hannover, Hannover, Germany

**Keywords:** Community-acquired pneumonia, Immunoglobulin therapy, Outcome, Biomarkers, Inflammation, Immunomodulation

## Abstract

**Background:**

The phase II CIGMA trial performed in 160 patients with severe community-acquired pneumonia (sCAP) found treatment with trimodulin (human polyvalent immunoglobulin [Ig]: ~ 23% IgM, ~ 21% IgA, ~ 56% IgG) was associated with a lower mortality in those patients with elevated baseline serum levels of C-reactive protein (CRP) and/or subnormal IgM.

**Methods:**

In this post hoc analysis, the pharmacodynamic effects of trimodulin treatment (182.6 mg/kg/day for 5 days) were investigated on Ig replenishment, cellular markers of inflammation (absolute neutrophil [ANC] and lymphocyte [ALC] count, neutrophil-to-lymphocyte ratio [NLR]), and soluble markers of inflammation (procalcitonin [PCT] and CRP). The impact of these pharmacodynamic effects on mortality was also evaluated.

**Results:**

Compared with healthy subjects, baseline serum levels of IgM, IgG, and ALC were significantly lower, and ANC, NLR, PCT and CRP significantly higher in sCAP patients (*p* < 0.0001). Low Ig concentrations increased with trimodulin. Normalization of ANC (analysis of variance [ANOVA] *p* = 0.016) and PCT (ANOVA *p* = 0.027) was more rapid with trimodulin compared with placebo. These and other effects were more evident in patients with low baseline IgM levels. Normalization of PCT and CRP levels was both steadier and faster with trimodulin treatment. In patients with low baseline ALC, trimodulin was associated with a lower 28-day all-cause mortality rate (14.5% vs 32.1% in placebo, *p* = 0.043) and more ventilator-free days ([VFD]; median VFD: 3.5 vs 11 in placebo, *p* = 0.043). These numerical differences were greater if baseline IgM was also low (low ALC, low IgM: 8.1% mortality vs 34.1% placebo, *p* = 0.006; 3 VFD vs 15 VFD, *p* = 0.009, respectively). Results were consistent in patients with high baseline CRP (low ALC, high CRP: 10.9% mortality vs 34.1% placebo, *p* = 0.011).

**Conclusions:**

This post hoc pharmacodynamic analysis of a blinded phase II trial suggests that trimodulin compensates for, and more rapidly modifies, the dysregulated inflammatory response seen in sCAP patients. Trimodulin was associated with significantly lower mortality and more VFD in subgroups with high CRP and low ALC. This effect was particularly marked in patients who also had low baseline IgM values. These findings require confirmation in prospective trials.

**Supplementary Information:**

The online version contains supplementary material available at 10.1186/s13054-023-04719-9.

## Background

Severe community-acquired pneumonia (sCAP) still carries high mortality and morbidity despite adequate antibiotic therapy [1, 2]. An excessive underlying inflammatory host response results in impaired gas exchange and distant organ failure, with an increased risk of death [2]. CAP guidelines do not currently recommend use of adjunctive immunomodulatory/anti-inflammatory interventions other than a suggestion to consider corticosteroid therapy if shock coexists [3, 4].

The host immune response includes recruitment of neutrophils to the site of infection which, together with immunoglobulins (Ig) and the complement system, supports phagocytosis of pathogens and removal of damaged host cell constituents [5]. However, excess neutrophil activation, in combination with low Ig concentrations and high levels of activated complement factors (for example, C5a), induces over-production of inflammatory cytokines, reactive oxygen species, proteases and neutrophil extracellular traps (NETs). These may themselves result in local tissue damage and even progression to systemic inflammation and organ dysfunction [6, 7]. Neutrophilia, lymphopenia and, accordingly, a high neutrophil-to-lymphocyte ratio (NLR) are markers of disease severity and worse outcomes in severe infections including sCAP [6, 8].

Hypogammaglobulinemia can occur in sCAP [9–12] from a combination of increased Ig consumption, in part related to excess neutrophil activation, and decreased Ig production related to lymphopenia [13–16]. B-cell maturation and clonal expansion to either plasma B-cells or long-lived memory B-cells (that express large amounts of antigen-specific Ig molecules) are also diminished [17]. Depletion in serum IgM and/or IgG, and sometimes IgA, are also related to disease severity and mortality [18–20]. This association is seen with severe viral lung infections [11, 21, 22], bacterial infections and septic shock [23–27].

Immunoglobulin-based therapy can both augment the host’s anti-microbial activity and modify the dysregulated immune response [28–30]. Trimodulin, a human plasma-derived native polyvalent IgM/IgA-enriched Ig preparation (~ 23% IgM, ~ 21% IgA, and ~ 56% IgG), was previously investigated in 160 patients with sCAP requiring invasive mechanical ventilation (IMV) in the prospective, placebo-controlled, phase II CIGMA trial [12]. In post hoc exploratory analyses, significant reductions in mortality were seen with trimodulin in subsets of patients with a hyperinflammatory response at baseline (indicated by a high C-reactive protein [CRP] level and/or low IgM level) [12]. These findings were supported by in vitro laboratory studies showing that trimodulin dampened secretion of infection-induced pro- and anti-inflammatory cytokines such as interleukin (IL)-6, tumor necrosis factor-alpha (TNF-α) and IL-1β [31–33].

The hypothesis underpinning this additional post hoc analysis of the phase II CIGMA trial was that the observed survival benefit with trimodulin results from beneficially modulating the dysregulated inflammatory response in sCAP patients. Our aim was to evaluate the pharmacodynamic effects of trimodulin on different cellular and soluble inflammation markers associated with sCAP. The impact of trimodulin on mortality in patient subgroups with different baseline levels of dysregulated inflammatory markers was also assessed.

## Methods

### Trial design and patient populations

This was a post hoc analysis of clinical data from the randomized, double-blind, placebo-controlled phase II CIGMA trial conducted in hospitals in Germany, Spain and the UK (EudraCT 2010-022380-35). Adult patients (19–93 years, *n* = 160) with sCAP requiring IMV received intravenous infusions of either a human polyvalent IgM/IgA-enriched Ig preparation at a dose of 3.65 mL/kg bodyweight/day (182.6 mg/kg trimodulin, 5% solution [BT086]; Biotest AG, Dreieich, Germany) or 3.65 mL/kg bodyweight/day placebo (36.5 mg/kg human albumin, 1% solution, Biotest AG, Dreieich, Germany) over five consecutive days [12]. The outcome of the trial has been reported previously [12].

To compare baseline levels of cellular and soluble markers of inflammation in sCAP patients enrolled in the CIGMA trial with those of healthy subjects, data were included from a phase I study conducted in Germany involving 24 healthy adults (20–45 years) (EudraCT 2007-005855-41). This study assessed the pharmacokinetics (PK) (IgM, IgA, and IgG), safety, and tolerability of trimodulin. The PK data from this study have been reported previously [34–36].

### Laboratory assessments in sCAP patients

In the CIGMA trial, absolute lymphocyte (ALC) and neutrophil count (ANC) in blood, and levels of CRP and procalcitonin (PCT) in serum, were determined at various timepoints before (baseline), during and after treatment with trimodulin or placebo. Measurements were performed at laboratories local to each site. Serum IgM, IgA, and IgG concentrations were assessed centrally (SGS Analytics Germany GmbH, Berlin, Germany) in a PK subgroup (*n* = 21), with sampling performed pre-treatment (baseline, *n* = 14), pre-dose, 4 h after start of infusion and at the end of infusion on Days 1–4 (*n* = 18–20). Additional samples were taken on Day 5 (pre-dose and at 2, 4 and 7 h after the end of infusion), on Days 6, 7, 14, 21, and at Day 28 or ICU discharge. For all other patients (*n* = 139), Ig concentrations were assessed only at baseline from retention samples.

### Evaluation of immune status

In the phase I study in healthy subjects, pre-treatment immune status was evaluated at the cellular level (ALC, ANC, NLR, monocyte and platelet counts) by measurement of soluble markers of inflammation (serum CRP and albumin), and Ig levels (IgM, IgA, and IgG).

In the phase II CIGMA trial, ALC, ANC, NLR, monocyte and platelet counts and serum CRP, PCT, albumin, IgM, IgA, and IgG were assessed pre-initiation of trimodulin (baseline). The course of these biomarkers was assessed during and shortly after treatment with trimodulin or placebo was concluded (up to Day 7).

### Subgroups

Patients enrolled in the CIGMA trial were grouped according to baseline levels of inflammatory markers. An overview of subgroups is presented in Table 1 and in Additional file 1: Fig. S1. Thresholds for these subgroups were in accordance with normal reference ranges for both cellular and soluble markers of inflammation (Tables 1 and 2). Subgroups were defined based on baseline levels either above (ANC^high^, NLR^high^, PCT^high^), below (ALC^low^, IgM^low^) or within (ANC^normal^) these reference ranges. The NLR^high^ subgroup comprised patients with both ALC^low^ and ANC^high^ at baseline (*n* = 65; median NLR 26.1; mean NLR 33.4). Subgroups with baseline thresholds ≥ 70 mg/L for CRP and ≤ 0.8 g/L for IgM were identified, as reported previously [12].

**Table 1:** Overview of subgroups for pharmacodynamic and efficacy assessments

Subgroups analyzed* (in addition to “All”)	Criteria
**Lymphocytes** (normal range: 1–4 × 10^9^/L) [37]	
ALC^low^	ALC < 1.0 × 10^9^/L (lymphopenia)
IgM^low^	IgM ≤ 0.8 g/L [12]
ALC^low^ + IgM^low^	As above
% of patients maintaining/reaching ALC^normal^ over time	ALC ≥ 1.0 × 10^9^/L
**Neutrophils**^**†**^ (normal range: 2.5–8.0 × 10^9^/L) [37]	
ANC^normal^	ANC < 8.0 × 10^9^/L
ANC^high^	ANC ≥ 8.0 × 10^9^/L (neutrophilia)
IgM^low^	IgM ≤ 0.8 g/L [12]
ANC^normal^ + IgM^low^	As above
ANC^high^ + IgM^low^	As above
% of patients maintaining ANC^normal^ over time	ANC < 8.0 × 10^9^/L
**NLR** (mild disease: 6 to < 9, moderate disease: 9 to < 18, critical disease: ≥ 18) [38]	
NLR^high^	Combined ALC^low^ + ANC^high^ resulting in NLR > 9
NLR^high^ + IgM^low^	As above
% of patients maintaining/reaching mild disease over time	NLR < 9.0
**PCT** (normal range/non-infected: ≤ 0.1 µg/L) [39]	
PCT^high^	PCT ≥ 2 µg/L (systemic bacterial infection threshold)
PCT^≤10 µg/L^	PCT ≤ 10 µg/L (up to severe systemic bacterial infection)
PCT^≤10 µg/L^ + IgM^low^	As above
PCT^≤10 µg/L^ + NLR^high^	As above
PCT^≤10 µg/L^ + CRP^high^	As above and below
**CRP** (normal range: 0–10 mg/L) [40]	
CRP^high^	CRP ≥ 70 mg/L
CRP^low^	CRP < 70 mg/L
CRP^high^ + IgM^low^	As above
CRP^high^ + ALC^low^	As above
CRP^high^ + NLR^high^	As above

*ALC* absolute lymphocyte count, *ANC* absolute neutrophil count, *CRP* C-reactive protein, *Ig* immunoglobulin, *NLR* neutrophil-to-lymphocyte ratio, *PCT* procalcitonin

*According to levels at baseline for sCAP patients in the phase II CIGMA trial;

^**†**^None of the sCAP patients had severe neutropenia (ANC < 0.5 × 10^9^/L)

**Table 2:** Demographics and baseline characteristics

Parameter	Normal reference range [37–43]	sCAP patients (phase II CIGMA) *N* = 160	Healthy subjects (phase I) *N* = 24	*p*-value^a^
Male, *n* (%)	n/a	113 (70.6)	10 (41.7)	n/d
Race, *n* (%)				
Caucasian	n/a	154 (96.3)	22 (91.7)	n/d
African		4 (2.5)	0	
Asian		1 (0.6)	2 (8.3)	
Other (Latin/Hispanic)		1 (0.6)	0	
Country, *n* (%)				
Germany	n/a	38 (23.8)	24 (100)	n/d
Spain		108 (67.5)	0	
UK		14 (8.8)	0	
Age (years), mean (SD)	n/a	65 (15)	30 (7)	n/d
BMI (kg/m^2^), mean (SD)	n/a	26.2 (4.9)	23.6 (3.2)	n/d
IgM (g/L)				
*n*	0.4–2.3 [41]	160	24	< 0.001
Median (IQR)		0.57 (0.38–0.88)	1.0 (0.8–1.4)	
IgG (g/L)				
*n*	7–16 [41]	160	24	< 0.001
Median (IQR)		6.6 (4.6–8.7)	9.9 (8.5–12.4)	
IgA (g/L)				
*n*	0.7–4.0 [41]	160	24	0.360
Median (IQR)		2.1 (1.4–3.2)	2.0 (1.6–2.5)	
Neutrophils (10^9^/L)				
*n*	2.5–8.0 [37]	150	24	< 0.001
Median (IQR)		11.5 (6.0–17.1)	3.7 (3.2–4.7)	
Lymphocytes (10^9^/L)				
*n*	1.0–4.0 [37]	150	24	< 0.001
Median (IQR)		0.69 (0.42–1.0)	1.6 (1.3–2.3)	
NLR				
*n*	0.8–3.5 [42]	149	24	< 0.001
Median (IQR)		16.1 (8.3–27.3)	2.1 (1.5–3.1)	
Monocytes (10^9^/L)				
*n*	0.1–0.7 [37]	155	24	0.468
Median (IQR)		0.51 (0.23–0.90)	0.44 (0.35–0.56)	
Platelets (10^9^/L)				
*n*	150–450^[[43]]^	160	24	0.516
Median (IQR)		202 (149–277)	225 (182–259)	
CRP (mg/L)				
*n*	0–10 [40]	155	24	< 0.001
Median (IQR)		230 (113–333)	0.1 (0.1–0.2)	
PCT (µg/L)				
*n*	≤ 0.10 [39]	145	n/d	n/d
Median (IQR)		5.1 (0.91–20.5)		
Albumin (g/L)				
*n*	35–55 [43]	125	24	< 0.001
Median (IQR)		27.0 (24.1–31.0)	47.0 (46.0–50.0)	

*BMI* body mass index, *CRP* C-reactive protein, *Ig* immunoglobulin, *IQR* interquartile range, *NLR* neutrophil-to-lymphocyte ratio, *PCT* procalcitonin, *sCAP* severe community-acquired pneumonia, *SD* standard deviation, *n/a* not applicable, *n/d* not determined, *UK* United Kingdom

^a^The Shapiro–Wilk normality test did not show a normal distribution for any of the laboratory parameters measured in sCAP patients and for some of the parameters measured in healthy subjects (CRP, IgM, and monocytes). Accordingly, unpaired, nonparametric Mann–Whitney testing was performed to determine the differences between sCAP and healthy subjects for all parameters

Baseline PCT levels were significantly lower in trimodulin-treated sCAP patients (*n* = 75) than in placebo-treated patients (*n* = 70) (median interquartile range [IQR] 2.3 µg/L [0.7, 10.4] vs 8.7 µg/L [1.8, 30.6]; *p* = 0.0016) (Additional file 1: Fig. S2). This was most likely caused by an imbalance between treatment groups in the number of patients infected with *Streptococcus* spp*.* (15 trimodulin vs 23 placebo [12]). Eleven of these patients had high PCT levels (> 50 µg/L [threshold representing 2 standard deviation (SD) from the mean PCT^All^ value]), among which 5/10 were in the placebo and 1/1 was in the trimodulin group. To account for this disparity, a subgroup with baseline PCT ≤ 10 µg/L (*n* = 92) was defined to aid comparison of treatment effects within the trimodulin and placebo groups (PCT^≤10 µg/L^; trimodulin median [IQR] 1.4 µg/L [0.5, 4.1]; placebo: 1.9 µg/L [0.5, 4.7]). Given that the intention is for trimodulin to be used early in the disease course, and that a level of PCT > 10 µg/L is indicative of advanced severe bacterial infection, only the PCT^≤10 µg/L^ subgroup was used for analysis.

### Assessment of the impact of trimodulin on mortality and ventilator-free days

The impact of trimodulin on 28-day all-cause mortality and ventilator-free days in the different subgroups was compared with those in the placebo subgroups using Chi-square tests and one-tailed Mann–Whitney test.

### Statistical analysis

Fold changes were assessed by comparing baseline values (Day 0, pre-treatment) with those measured on post-infusion days. NLR was determined by dividing ANC by ALC.

Descriptive statistical analyses were performed using SAS (Version 9.4) and GraphPad Prism Software (Version 6.07). Pairwise comparisons of concentrations or cell numbers between trimodulin and placebo were made using unpaired Wilcoxon–Mann–Whitney tests. If not otherwise specified, categorical variables were analyzed by two-sided Chi-square test for proportions, and continuous variables by two-sided Student’s *t*-test. Repeated measures analysis of variance (ANOVA) assessed between-subject effects of treatment over time (from baseline to Day 7). All patients were included within the analysis of time-to-first change above or below a threshold. If no value above or below the threshold was available over time, Day 21 was assumed. The significance level was set throughout to *p* < 0.05, if not stated otherwise.

These post hoc exploratory analyses were not adjusted for Type I error and are thus prone to an inflated error rate due to multiple comparisons. Our study aims were to identify potential predictive biomarkers and mechanistic clues as to why some patients benefited whereas others did not.

## Results

### Immune profile in patients with sCAP and in healthy adults

Demographics and baseline characteristics of sCAP patients (CIGMA trial) and healthy subjects (phase I study) are presented in Table 2. Compared with healthy adults and normal reference ranges, adult patients with sCAP had significantly lower values of IgM, IgG, lymphocytes and serum albumin, and significantly higher values for neutrophils, NLR, CRP and PCT. Monocytes, platelet count and IgA were within normal ranges and similar in both groups (Table 2).

In the CIGMA trial, corticosteroid use was balanced between trimodulin and placebo groups with 61/81 (75%) and 64/79 (81%) exposed, respectively. The same was found for other drugs with immunomodulatory activity used to treat sCAP or any other underlying inflammatory disease (Additional file 1: Table S1).

### Impact of trimodulin on immunoglobulin status

**Fig. 1:**
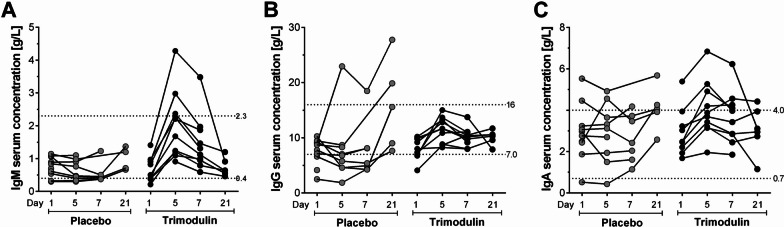
Before-and-after plots of Ig serum concentrations in sCAP patients treated with placebo or trimodulin. The kinetics of immunoglobulins during infection was investigated in serum of patients with sCAP in the PK subset (*n* = 21). On day 5, in sCAP patients treated with trimodulin, **A** median levels of IgM increased from 0.5 to 1.9 g/L, **B** median levels of IgG increased from 8.0 to 11.7 g/L, and **C** median levels of IgA increased from 2.5 to 3.8 g/L. Baseline is the pre-dose level measured before start of infusion on Day 1 (trimodulin *n* = 9, placebo *n* = 10) while Day 5 is the post-dose level taken after the infusion ended (trimodulin *n* = 10, placebo *n* = 9). On Day 7 data were available for trimodulin *n* = 10, placebo *n* = 7, and on Day 21 for trimodulin *n* = 6, placebo *n* = 5 patients. Complete before-and-after-plots were possible for five trimodulin- and four placebo-treated patients (indicated by linked Day 1, 5, 7 and Day 21 data points). Dotted lines indicate normal reference ranges (Table 2, [41]). *Ig* immunoglobulin, *PK* pharmacokinetics, *sCAP* severe community-acquired pneumonia

Ig concentrations were analyzed in the PK subgroup (*n* = 21) within the CIGMA trial. Treatment of sCAP patients with trimodulin replenished levels of IgM and IgG from the lower normal range to the mid–upper level of normal on day 5. This was not generally observed in patients in the placebo group; IgG levels fluctuated and IgM concentrations remained much lower over the 21-day disease course (Fig. 1A, B). IgA levels, which were not depleted in sCAP patients at baseline, were all elevated to the upper range of normal after trimodulin treatment on day 5 but fluctuated in the placebo group (Fig. 1C).

### Impact of trimodulin on cellular responses in sCAP patients

Given the survival benefit associated with trimodulin use in sCAP patients with low baseline IgM levels [12], the effects of trimodulin on cellular and soluble markers of immune status and inflammation were investigated in both the overall population and in the subgroup of patients with low IgM.

#### Absolute lymphocyte count

**Fig. 2:**
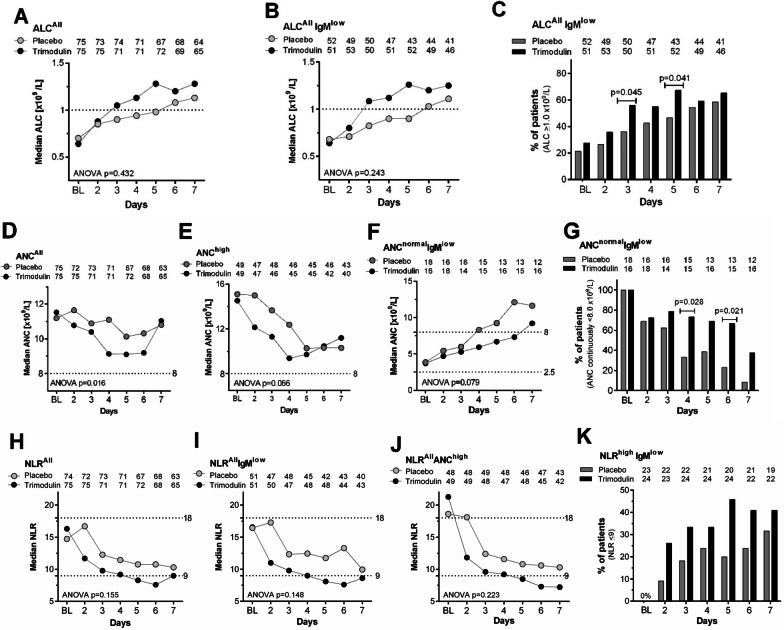
Cellular pharmacodynamic responses in sCAP patients modulated by trimodulin. **A** Time course of ALC in all patients (ALC^All^) during (Day 1–5) and after treatment (Day 6–7) with trimodulin or placebo. The numbers of subjects per group and per day are indicated above the graph. The dotted line represents the threshold for normal ALC (≥ 1.0 × 10^9^/L). **B** As in A, for the subgroup of patients who also had low baseline IgM (≤ 0.8 g/L) (ALC^ALL^ IgM^low^ [trimodulin, median IgM 0.46 g/L and median ALC 0.64 × 10^9^/L; placebo, median IgM 0.44 g/L and median ALC 0.68 × 10^9^/L). **C** Bar graph showing the percentage of patients at different timepoints with normal ALC. **D** Time course of neutrophil levels in all patients treated with trimodulin or placebo. The dotted line represents the threshold for neutrophilia (≥ 8.0 × 10^9^/L, ANC^high^). **E** Time course of neutrophil levels in the subgroup of patients in the ANC^high^ subgroup during and after treatment with trimodulin or placebo. **F** As in E for the ANC^normal^ IgM^low^ subgroup of patients with low IgM. **G** Bar graph showing the proportion of patients in the ANC^normal^ IgM^low^ subgroup where ANC remains normal over time. **H** Time course of the NLR in patients treated with trimodulin or placebo. Dotted lines: NLR values between 9 and 18 reflect moderate illness, whereas higher values reflect severe/critical disease and lower values mild illness [38]. **I** NLR as in H for the IgM^low^ subgroup of patients. **J** NLR as in H for the ANC^high^ subgroup. **K** Bar graph showing the proportion of patients in the NLR^high^ IgM^low^ subgroup returning to NLR levels < 9 during and after treatment with trimodulin or placebo. To analyze statistical differences in the overall course of responses ANOVA was used in (**A**–**B**, **D**–**F**). On each treatment day the two-tailed Chi-square test was used to assess the statistical significance of differences between the two treatment groups in the % of patients with lymphocytes returning to ≥ 1.0 × 10^9^/L (**C**), neutrophils remaining at < 8.0 × 10^9^/L (**G**) or an NLR returning to < 9 (**K**). For days where differences were statistically significant, *p*-values are given above the bars. For further details see Additional file 1: Fig. S5. *ALC* absolute lymphocyte count, *ANC* absolute neutrophil count, *ANOVA* analysis of variance, *BL* baseline, *Ig* immunoglobulin, *NLR* neutrophil-to-lymphocyte ratio

Lymphocyte data were available for 150 patients (75 trimodulin; 75 placebo). Baseline values of ALC in sCAP patients did not differ between trimodulin and placebo groups (Additional file 1: Fig. S3A). ALC fluctuated over time in individual patients treated with placebo (Additional file 1: Fig. S4). By contrast, ALC remained largely within the normal range in trimodulin-treated patients. Lymphocyte data were available in 150 patients (ALC^All^, trimodulin *n* = 75; placebo *n* = 75). Median ALC was first restored to the normal range by Day 3 with trimodulin and by Day 6 in the placebo group (Fig. 2A). The impact was similar in patients with low IgM at baseline (ALC^All^ IgM^low^ subgroup, *n* = 103) (Fig. 2B). The proportion of patients with a normal ALC was significantly higher on Days 3 and 5 in the ALC^All^ IgM^low^ subgroup treated with trimodulin (Fig. 2C).

Baseline lymphopenia (ALC^low^) was recorded in 55 (73.3%) of the 75 trimodulin-treated patients and 56 (74.7%) of 75 placebo patients. The time to first achieve normal values in ALC^low^ patients did not differ significantly [Wilcoxon-test *p* = 0.16] between trimodulin (median: 3 days) and placebo (5 days) (data not shown).

#### Absolute neutrophil count

Neutrophil data were available for 150 patients (75 trimodulin; 75 placebo). Baseline values of ANC in sCAP patients did not differ between trimodulin and placebo groups (Additional file 1: Fig. S3B). Overall values (ANC^All^) differed significantly over time between the two treatment groups (Fig. 2D). Baseline neutrophilia (ANC^high^) was present in 49 patients in each group. Patients receiving trimodulin had a faster, albeit non-significant (*p* = 0.066), return toward the normal range (Fig. 2E). By contrast, in both patients with normal baseline ANC (ANC^normal^ [data not shown]) and in the subset with a low IgM (ANC^normal^ IgM^low^ [Fig. 2F]), median ANC increased to above normal by Day 4 in the placebo group, whereas this was delayed in the trimodulin group until Day 7. In the ANC^normal^ IgM^low^ subgroup, the proportion of patients with a normal ANC was significantly higher on Days 4 and 6 of treatment with trimodulin (Fig. 2G).

#### Neutrophil-to-lymphocyte ratio

NLR was calculated in all patients (NLR^All^) with available baseline ANC and ALC data (75 trimodulin; 74 placebo). An initial rise was seen in the placebo group with a fall thereafter, whereas NLR fell immediately in trimodulin-treated patients (Fig. 2H). These differences were more pronounced, albeit non-significantly, in the NLR^All^ IgM^low^ subset (Fig. 2I). A similar trend was seen in the ANC^high^ subgroup (Fig. 2J). The proportion of patients in the NLR^high^ IgM^low^ subgroup attaining an NLR level ≤ 9 was up to threefold higher in the trimodulin group (Fig. 2K). However, due to the small number of patients in this subgroup, between-group differences were not statistically significant.

### Impact of trimodulin on soluble markers of inflammation in sCAP patients

#### Cytokines

Although no cytokine data were available from patients in the phase II CIGMA trial, trimodulin has been found to downregulate secretion of IL-6, TNF-α and IL-1β by endotoxin-stimulated immune cells in vitro (Additional file 1: Fig. S6). An indirect effect on CRP and PCT was thus predicted in patients treated with trimodulin.

#### C-reactive protein

**Fig. 3:**
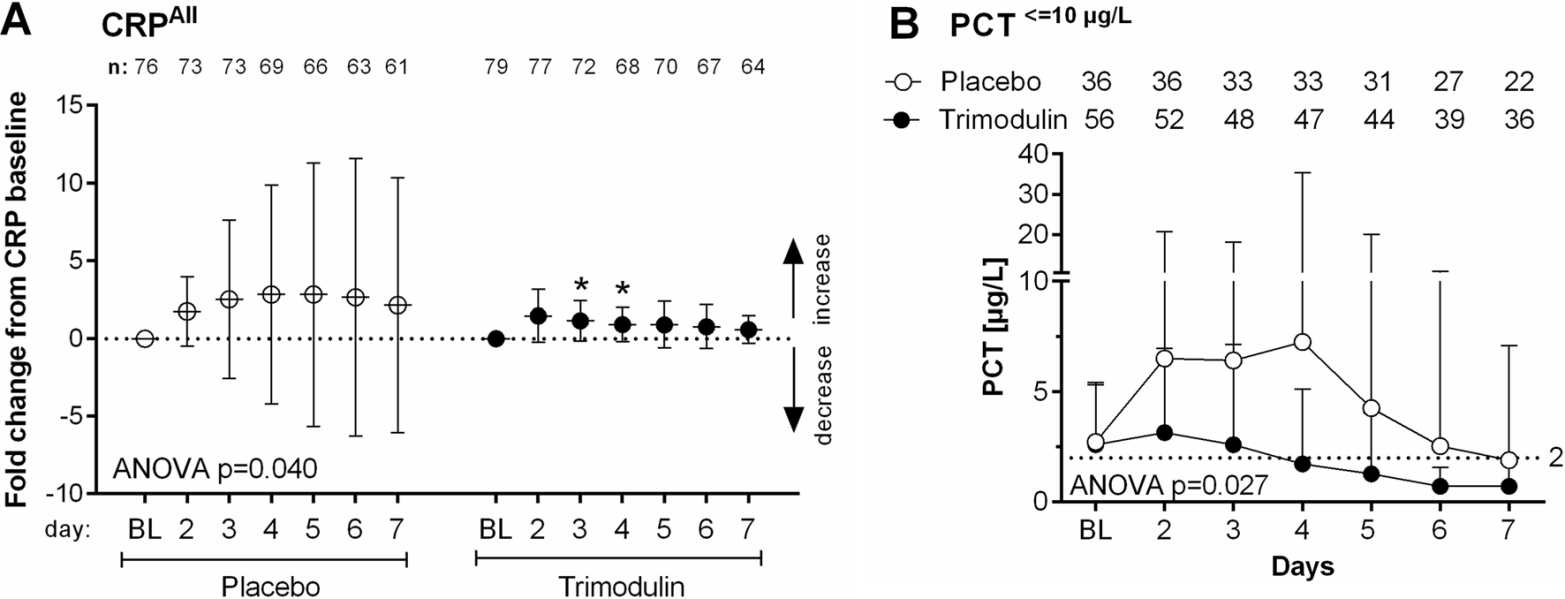
Temporal changes in C-reactive protein and procalcitonin plasma concentrations. **A** Mean fold change from baseline CRP levels in all patients with an available value was calculated per patient. The mean ± SD baseline for the trimodulin group was 222 ± 129 mg/L and 227 ± 147 mg/L for the placebo group. The dotted line (0) represents the no-change level. Patient numbers decreased after Day 2 due to discharge or death (indicated at the top of the figure). Repeated measures ANOVA assessed between-subject effects of treatment over time (from baseline to Day 7). *Indicates *p* < 0.05 (*t*-test with Welch’s correction: Day 3, *p* = 0.0281; Day 4, *p* = 0.0291). **B** PCT (mean + SD) in the subgroup of patients with baseline PCT ≤ 10 µg/L (*n* = 92). A significant difference between overall PCT concentration-time profiles was found between groups, but no statistically significant differences were observed at single timepoints. *ANOVA* analysis of variance, *BL* baseline, *CRP* C-reactive protein, *PCT* procalcitonin, *SD* standard deviation

CRP data were available for 155 patients at baseline (79 trimodulin; 76 placebo). Baseline values of CRP in sCAP patients did not differ between trimodulin and placebo groups (Additional file 1: Fig. S3C), nor over time. Time-concentration profiles up to Day 21 in 10 patients in each group revealed substantial fluctuation in CRP levels with placebo. In patients receiving trimodulin, a steadier reduction was observed with levels remaining low after the end of treatment on Day 5 (Additional file 1: Fig. S7A). The fold change from baseline values also differed significantly between groups (Fig. 3A). To investigate a potential confounding effect of steroid treatment, which is known to affect CRP levels, the effect of trimodulin on CRP was additionally analyzed in the subset of patients who did not receive steroids during the trial (28 trimodulin; 20 placebo). The course of fold change from baseline (Additional file 1: Fig. S7B) was consistent with that observed in all patients (Fig. 3A) although no significant difference was observed between groups due to their smaller size. This indicates that the effect observed with trimodulin was independent of steroid treatment during the trial (Additional file 1: Fig. S7B).

#### Procalcitonin

PCT data were available for 92 patients with a baseline concentration of PCT ≤ 10 μg/L (56 trimodulin; 36 placebo). Baseline PCT levels were similar between groups (mean ± SD: 2.6 ± 2.8 µg/L placebo, 2.7 ± 2.6 µg/L trimodulin). As with CRP, time-concentration profiles of PCT in 10 patients per group showed more fluctuation with placebo compared with trimodulin (Additional file 1: Fig. S7C).

A significant difference was observed between the PCT time-concentration profiles for the trimodulin and placebo groups (Fig. 3B). An initial increase in PCT levels was observed in placebo-treated patients on Days 2–4 followed by a slow decrease (Fig. 3B). In contrast, PCT levels in the trimodulin group decreased below the threshold for systemic infection (< 2 µg/L) and toward normal (≤ 0.1 µg/L) more rapidly and steadily compared with placebo (Fig. 3B, Additional file 1: Fig. S7D). Similar trends were apparent in the PCT^≤10 µg/L^ IgM^low^ subset (Additional file 1: Fig. S7E) and in the fold change from baseline (Additional file 1: Fig. S7F). The time to reach a PCT threshold < 2 µg/L was significantly (*p* = 0.019) shorter with trimodulin treatment (8.2 days) compared with placebo (11.6 days).

### Association between indicators of dysregulated inflammatory responses and mortality in placebo-treated sCAP patients

Most non-survivors in the placebo group (*n* = 22) had indicators of dysregulated inflammatory responses at baseline (including low ALC [in 82% of non-survivors], high ANC [64%], high NLR [91%], increased CRP [86%] and increased PCT [84%]) (Additional file 1: Fig. S8A, B). Low IgM and IgG levels were more commonly seen in placebo group non-survivors [77% and 68%, respectively), but no association was evident between IgA levels and mortality (Additional file 1: Fig. S8C).

### Impact of trimodulin on mortality in sCAP patients with indicators of dysregulated inflammatory responses

#### Cellular markers of inflammation

**Fig. 4:**
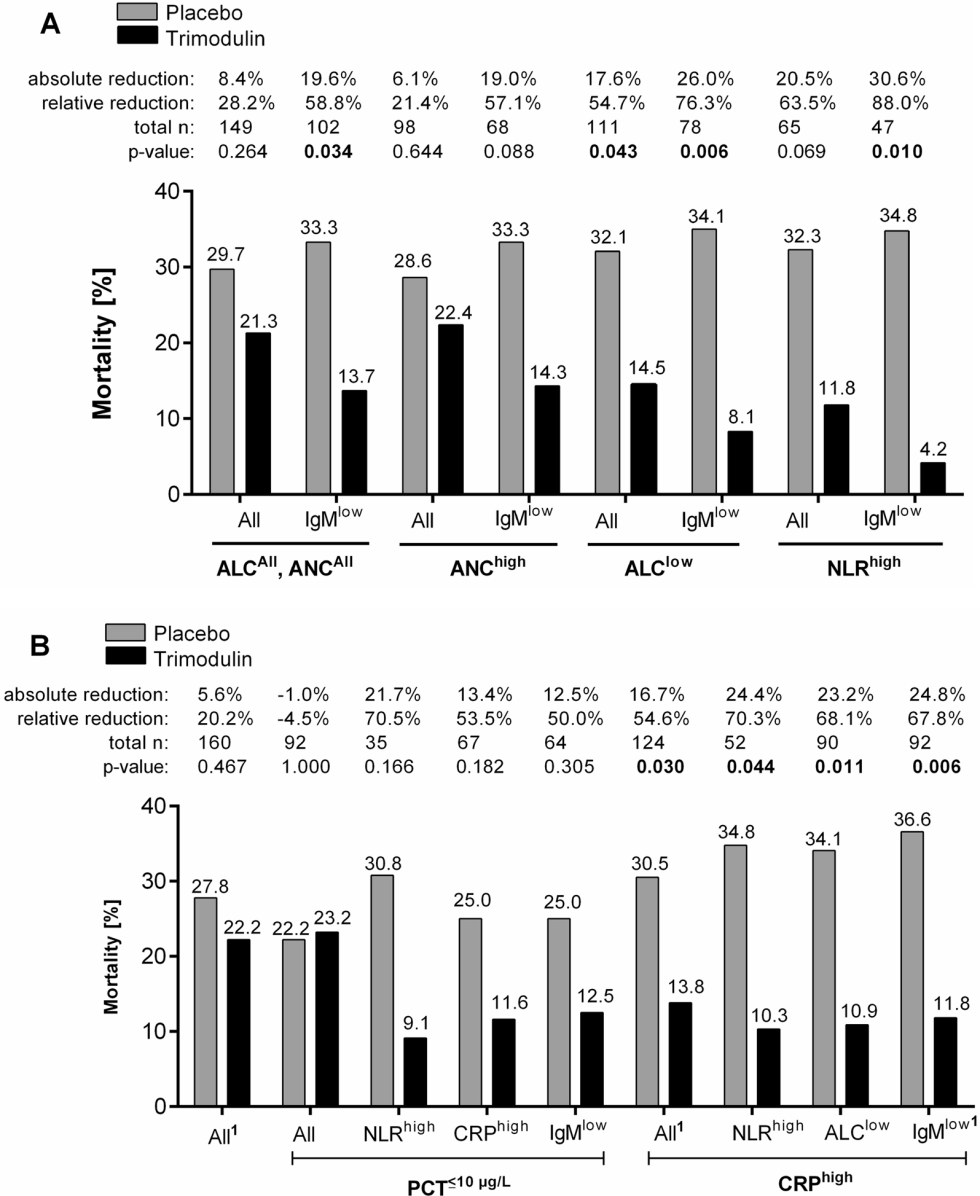
Mortality rates in sCAP patients treated with trimodulin or placebo. **A** Mortality in sCAP patients with IgM deficiency, combined with an impaired cellular immune status. **B** Mortality in sCAP patients with low IgM, neutrophilia, or lymphopenia combined with a hyperinflammatory status (PCT ≤ 10 µg/L, or CRP ≥ 70 mg/L). *p*-values were calculated by Chi-square tests. Subgroup criteria are presented in Table 1. ^1^Previously published data from the CIGMA trial [12] and included here for reference and completeness. *ALC* absolute lymphocyte count, *ANC* absolute neutrophil counts, *CRP* C-reactive protein, *Ig* immunoglobulin, *NLR* neutrophil-to-lymphocyte ratio, *PCT* procalcitonin, *sCAP* severe community-acquired pneumonia

In the ANC^high^ subgroup, mortality rates in the trimodulin group were 6.1% lower (absolute reduction) compared with placebo. This effect was more pronounced (19.0%) in those with additional low baseline IgM (Fig. 4A). Mortality was 17.6% lower in the ALC^low^ subgroup given trimodulin (Fig. 4A), a difference more pronounced in patients who in addition had a low baseline IgM (26.0% absolute reduction). Accordingly, mortality in the NLR^high^ and NLR^high^ IgM^low^ subgroups was 20.5% and 30.6% lower, respectively, in trimodulin-treated patients compared with placebo (Fig. 4A).

#### Soluble markers of inflammation

In patients with PCT ≤ 10 µg/L, mortality rates were similar between treatment groups (Fig. 4B). However, mortality rates were markedly lower in patients treated with trimodulin in subgroups with additional raised cellular (NLR^high^) or soluble (CRP^high^) markers of inflammation, or low Ig (IgM^low^) levels (Fig. 4B).

Similarly, in those with a high baseline CRP, mortality rates were lower in patients treated with trimodulin in the three subgroups with NLR^high^, ALC^low^ and IgM^low^ (Fig. 4B). IgM^low^ appears to be the strongest predictor of a beneficial treatment effect from trimodulin (Fig. 4B).

Although a mortality reduction was already seen in patients treated with trimodulin with a high baseline CRP, an even stronger effect was observed in the three CRP^high^ subgroups with NLR^high^, ALC^low^ or IgM^low^ (Fig. 4B).

### Impact of trimodulin on ventilator-free days in sCAP patients with indicators of dysregulated inflammatory responses

Number of ventilator-free days (VFD) was the primary endpoint of the CIGMA trial. Results from the primary endpoint analysis in the different subgroups revealed that the median number of VFD was higher, albeit not significantly so, in trimodulin-treated patients compared with placebo in subgroups with either CRP^high^, IgM^low^ or ALC^low^ at baseline (Table 3). This benefit of trimodulin treatment was, however, significant in patients with combined ALC^low^ IgM^low^ at baseline.

**Table 3:** Ventilator-free days in patients treated with trimodulin or placebo

Subgroup	Number of patients (% of total per arm)	Ventilator-free days		*p*-value*
		Placebo Median [IQR]	Trimodulin Median [IQR]	
All	P: 79 (100%) T: 81 (100%)	8.0 [0, 19.0]	11.0 [0, 20.0]	0.173^a^
CRP^high^	P: 59 (74.7%) T: 65 (80.2%)	4.0 [0, 19.0]	14.0 [0, 20.0]	0.043^a^
IgM^low^	P: 55 (69.6%) T: 56 (69.1%)	7.0 [0, 19.0]	15.0 [0.25, 20.0]	0.029^a^
ALC^low^	P: 56 (70.9%) T: 55 (67.9%)	3.5 [0, 18.8]	11 [0, 19.0]	0.043
CRP^high^ NLR^high^	P: 23 (29.1%) T: 29 (35.8%)	12.0 [0, 19.0]	15.0 [5, 21.0]	0.148
CRP^high^ + ALC^low^	P: 44 (55.7%) T: 46 (56.8%)	3.5 (0, 18.8]	11.5 [0, 20.0]	0.046
CRP^high^ + IgM^low^	P: 41 (51.9%) T: 51 (63.0%)	4.0 [0, 19.0]	15.0 [1.0, 20.0]	0.059^a^
NLR^high^ + IgM^low^	P: 23 (29.1%) T: 24 (29.6%)	7.0 [0, 19.0]	16.0 [9.0, 19.8]	0.057
ALC^low^ + IgM^low^	P: 41 (51.9%) T: 37 (45.7%)	3.0 [0, 18.0]	15.0 [4.5, 19.5]	**0.009**

*ALC* absolute lymphocyte count, *CRP* C-reactive protein, *IQR* interquartile range, *NLR* neutrophil-to-lymphocyte ratio, *P* placebo, *T* trimodulin

*One-sided Mann–Whitney test. The significance level is *p* < 0.025 (indicated in bold)

^a^Previously published data from the CIGMA trial [12]. Here, the *p*-value was calculated with a one-sided Wilcoxon rank sum test with continuity correction (0.5) and a significance level of 0.025

## Discussion

Patients with sCAP enrolled into the phase II CIGMA trial generally had low baseline levels of IgM and IgG, a finding observed previously in patients with sCAP and sepsis [24–27, 44]. The magnitude of abnormality of immune and inflammatory markers at baseline—lymphopenia, neutrophilia, a high NLR, elevated levels of CRP and PCT, and low levels of IgG and IgM—was associated with worse outcomes. This finding reflects previous studies that have also identified an increased mortality risk in sCAP patients with hyper-inflammation and a more dysregulated immune response [1, 2, 45, 46].

Large variations in the levels of inflammatory markers observed over time in the placebo-treated patients are also consistent with the occurrence of alternating hyper- and hypo-inflammatory states previously reported during severe infection [44]. A steady and more rapid modulation of these inflammatory and immune markers was observed in patients receiving trimodulin, a human polyvalent immunoglobulin containing ~ 23% IgM, ~ 21% IgA, ~ 56% IgG.

Trimodulin thus seems to be able to normalize immune dysregulation and reduce inflammation more rapidly in this patient population. This is also reflected in the mortality rates observed in the trimodulin- and placebo-treated groups where numerical differences were greater in those patients with more extreme immune/inflammatory values, i.e., ALC^low^, NLR^high^, CRP^high^. Notably, this between-group mortality difference was accentuated in those patients with a low baseline IgM level. Although these mortality data should be interpreted with caution given the low patient numbers in the subgroups, this finding does lend support to the rationale of using a preparation containing all three immunoglobulins rather than a standard intravenous preparation that comprises ≥ 95% IgG but little IgM. This post hoc analysis identified that the trimodulin regimen administered in the CIGMA trial was able to supplement IgM and IgG to levels at the upper normal range and stabilize IgG concentrations over the disease course. The benefit of supplementing IgA is less clear as baseline plasma IgA levels were not depleted. Nonetheless, this provides no insight as to the impact on alveolar IgA concentrations where IgA is active. Potentially, the higher IgA serum concentration after trimodulin treatment may supplement local concentrations and support the host defense against pathogens.

B- and T-cell lymphopenia are reported frequently in sCAP and sepsis patients [17, 47]. As shown previously [45] and confirmed in this *post hoc* analysis (Fig. 4A–B, Additional file 1: Fig. S8A), lymphopenia is associated with an increased risk of mortality in sCAP patients. Depletion of memory B-cells in sCAP has been described and may be linked to the toxic effects of antibiotics [17, 47]. B-cell lymphopenia may be a major cause of the Ig deficiency observed in sCAP patients. As lymphocyte subtypes were not determined in this analysis, more research is required. Supplementing hypogammaglobulinemia with trimodulin, particularly in the ALC^low^ IgM^low^ subgroup, was associated not only with a significant survival benefit but also with a significant increase in VFD.

In addition to lymphopenia, neutrophilia is also frequently seen in sCAP patients. Chemo-attractants secreted by damaged and inflamed tissue and locally activated immune cells culminate in cytokine release that further fuels the inflammatory response [6]. We advance two possible explanations for the rapid reduction in neutrophil counts observed with trimodulin (Fig. 2D, E, G). Firstly, trimodulin neutralizes both pathogen-associated (PAMPs) and damage-associated (DAMPs) molecular patterns, preventing Toll-like receptor signaling and thereby reducing cytokine and chemokine production; this, in turn, may decrease stimulation of the bone marrow to generate and release further neutrophils [48, 49]. Secondly, inflammation can result in delayed apoptosis and prolong neutrophil lifespan [50]). Although no cytokine data were available from the phase II CIGMA trial, we have found that trimodulin downregulates secretion of IL-6, TNF-α, and IL-1β by endotoxin-stimulated immune cells in vitro (Additional file 1: Fig. S6). Thus, if trimodulin is given in a timely manner, the inflammatory responses may be dampened and neutrophilia reversed more rapidly (Fig. 2F).

Changes in the NLR demonstrate the net impact of trimodulin treatment on ALC and ANC that were more rapidly normalized (Fig. 2H–K). High NLR values are related to an unfavorable prognosis in patients with sepsis [51]. Linking NLR to disease severity, trimodulin may shift patients from a state of critical disease to moderate illness within 2–3 days, and earlier than that seen in the placebo group.

In addition to the effects of trimodulin on the investigated cellular markers, the reduction in inflammation was also apparent through the enhanced and steady normalization of CRP and PCT levels (Fig. 3 and Additional file 1: Fig. S7). This complements studies in septic patients where use of the IgM/IgA-enriched preparation, Pentaglobin (12% IgM, 12% IgA, 76% IgG) was associated with consistently faster decreases in CRP and/or PCT compared with control [52–58].

In the phase II CIGMA trial, the largest mortality differences with trimodulin treatment compared with placebo were seen in the subgroup of patients with IgM^low^ and/or CRP^high^ at baseline [12]. The current analysis indicates an additional role for lymphopenia and a high NLR in contributing to mortality risk in these patients. Lymphopenia paired with low IgM levels and hyperinflammation may provide an advantage for the pathogen, promoting persistence, expansion, and replication [59]. In the current analysis, the lowest mortality relative to placebo was associated with the use of trimodulin in sCAP patient subsets with high CRP, low IgM, and/or lymphopenia and/or high NLR. This also corresponded with improvements in immune and inflammatory status.

### Study limitations

As this is a post hoc analysis, results should be used only for hypothesis generation. In addition to the small number of patients in some defined subgroups, larger prospective studies are warranted to confirm the findings. Laboratory values were partly assessed in different local and central laboratories and any slight variability in values was not considered. Reference ranges were not compared individually to those provided by the different laboratories, however values from the different labs were compared to more general reference ranges described in the literature [37–43]. As data from healthy subjects and sCAP patients were collected from separate studies conducted sequentially, this could have had an impact on comparability of data. Furthermore, the healthy subjects were not age- and comorbidity-matched with the sCAP patients. Finally, the low number of patients in the subgroups and large inter-patient differences resulted in high standard deviations and thus trends rather than non-significant changes.

## Conclusions

Results of immunomodulatory trials in patients with sCAP remain inconclusive [2]. Accordingly, no clear recommendation is provided in current American Thoracic Society/Infectious Diseases Society of America and European Respiratory Society/European Society of Intensive Care Medicine/European Society of Clinical Microbiology and Infectious Diseases/Latin American Thoracic Association guidelines for immunomodulatory treatment of sCAP, other than the consideration of corticosteroids if shock is present [3, 4]. Immune suppression may increase patient vulnerability to disease progression and/or nosocomial infection. This post hoc pharmacodynamic analysis of the CIGMA trial indicates that an IgM/IgA-enriched Ig solution is a promising therapeutic option for sCAP patients. It modified dysregulated inflammatory and immune responses, and this was associated with a survival benefit and reduced time on a ventilator, especially in those with significant immune and inflammatory changes. These results warrant further investigation of trimodulin in a randomized controlled trials of patients with sCAP with evidence of significant inflammation and Ig depletion.

### Supplementary Information

**Additional file 1**. **Table S1:** Immune modulating medications provided to sCAP patients before and/or during the trial. **Fig. S1:** Flowchart of the subgroups of sCAP patients analyzed in this post hoc study. **Fig. S2:** Difference in baseline PCT levels in the placebo and trimodulin groups. **Fig. S3:** Immune and inflammatory status in healthy subjects and sCAP patients at baseline. **Fig. S4:** Pharmacodynamic effect of trimodulin on ALC over time. **Fig. S5:** Cellular pharmacodynamic responses modulated by trimodulin in patients with sCAP. **Fig. S6:** Modulation of pro-inflammatory cytokine responses by trimodulin in vitro. **Fig. S7:** Modulation of CRP and PCT serum levels by trimodulin. **Fig. S8:** Dysregulated inflammatory responses in non-surviving sCAP patients in the placebo group.

## Data Availability

They are available from responsible author (CCH [corinacornelia.heinz@biotest.com]) upon reasonable request.

## Abbreviations

ALC: Absolute lymphocyte count
ANC: Absolute neutrophil count
ANOVA: Analysis of variance
BL: Baseline
CRP: C-reactive protein
DAMPs: Damage-associated molecular patterns
Ig: Immunoglobulin
IL: Interleukin
IMV: Invasive mechanical ventilation
IQR: Interquartile range
NETs: Neutrophil extracellular traps
NLR: Neutrophil-to-lymphocyte ratio
PAMPs: Pathogen-associated molecular patterns
PCT: Procalcitonin
PK: Pharmacokinetic
sCAP: Severe community-acquired pneumonia
SD: Standard deviation
TNF: Tumor necrosis factor
VFD: Ventilator-free days
