# Supplementary material for: The immunomodulating activity of trimodulin (polyvalent IgM, IgA, IgG solution): a post hoc analysis of the phase II CIGMA trial
Source: Crit Care. 2023 Nov 9;27:436. doi: 10.1186/s13054-023-04719-9 (PMC10634136; doi:10.1186/s13054-023-04719-9)
Supplement: Supplementary file 1 — Additional file 1. Table S1: Immune modulating medications provided to sCAP patients before and/or during the trial. Fig. S1: Flowchart of the subgroups of sCAP patients analyzed in this post hoc study. Fig. S2: Difference in baseline PCT levels in the placebo and trimodulin groups. Fig. S3: Immune and inflammatory status in healthy subjects and sCAP patients at baseline. Fig. S4: Pharmacodynamic effect of trimodulin on ALC over time. Fig. S5: Cellular pharmacodynamic responses modulated by trimodulin in patients with sCAP. Fig. S6: Modulation of pro-inflammatory cytokine responses by trimodulin in vitro. Fig. S7: Modulation of CRP and PCT serum levels by trimodulin. Fig. S8: Dysregulated inflammatory responses in non-surviving sCAP patients in the placebo group. [file 13054_2023_4719_MOESM1_ESM.docx]

# Supplementary file

The immunomodulating activity of trimodulin (polyvalent IgM, IgA, IgG solution): A *post-hoc* analysis of the phase II CIGMA trial

# Table S1: Immune modulating medications provided to sCAP patients before and/or during the trial.

| **Type of drug** (drug names) | **Placebo**  **(N=79)** | **Trimodulin**  **(N=81)** |
| --- | --- | --- |
| **Corticosteroids for systemic use** (budesonide, dexamethasone, hydrocortisone, methylprednisolone, prednisolone, beclometasone) | 64 (81.0%) | 61 (75.3%) |
| **Marcolides** (clarithromycin, azithromycin, roxithromycin, erythromycin) | 53 (67.1%) | 58 (71.6%) |
| **Fluoroquinolones** (levofloxacin, ciprofloxacin, moxifloxacin) | 51 (64.6%) | 50 (61.7%) |
| **Aspirin** (acetylsalicylic acid) | 25 (31.6%) | 25 (30.9%) |
| **Other anti-inflammatory drugs*** (diclofenac, aceclofenac, chondroitin, ibuprofen, dexketoprofen, hydroxychloroquine, tocilizumab, rofecoxib, naproxen, methotrexate, iloprost, allopurinol, colchicine) | 6 (7.6%) | 8 (9.9%) |

*These patients received anti-inflammatory/anti-rheumatic/anti-gout drugs without receiving additional corticosteroids.

# *Overview of subgroups of the CIGMA trial analyzed in this study*

| 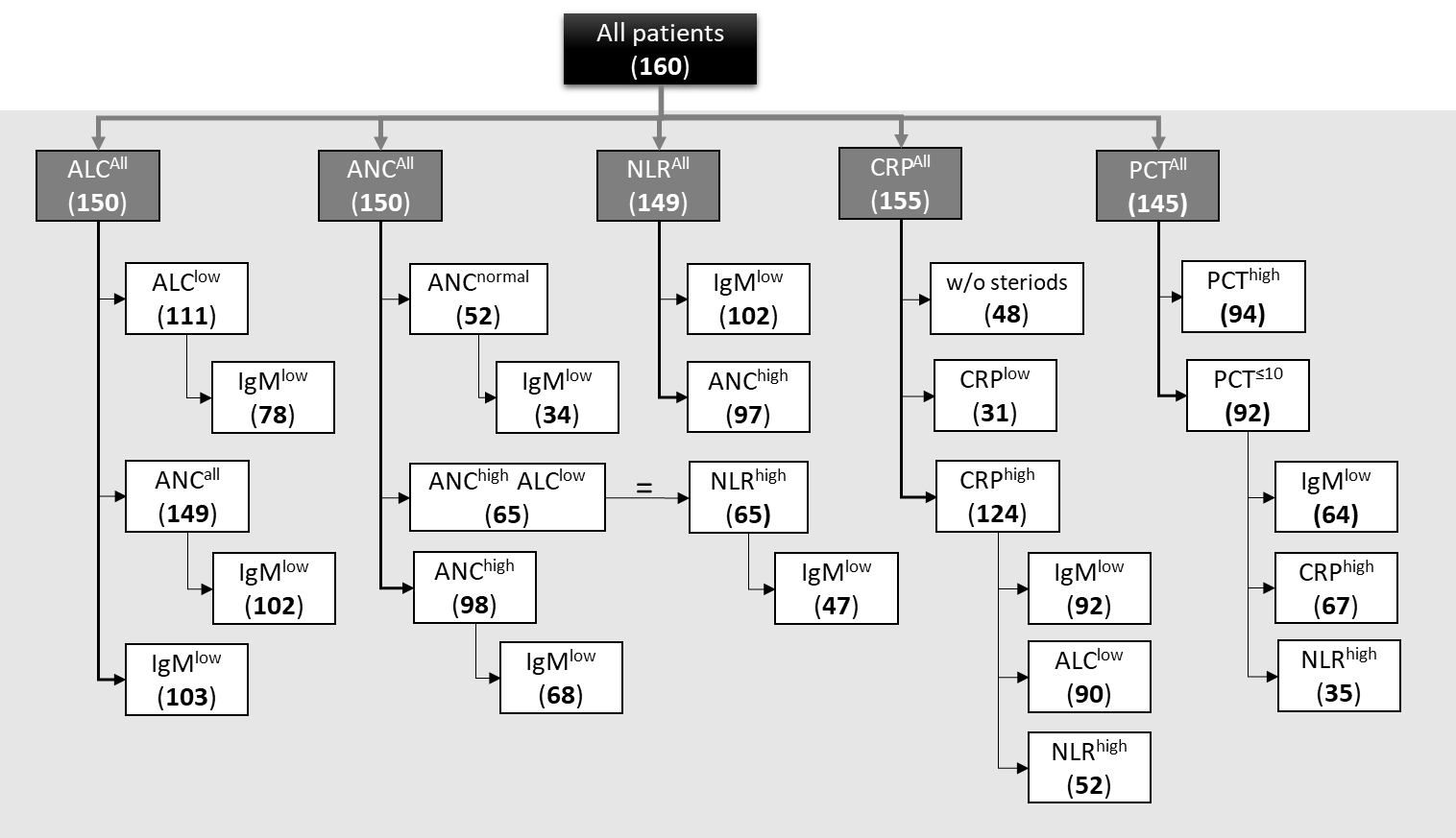 |
| --- |

**Fig. S1: Flowchart of the subgroups of sCAP patients analyzed in this *post-hoc* study.**

In this *post-hoc* study, additional subgroups were analyzed as shown. The number of patients per subgroup is indicated in bold in brackets. In addition to mortality and VFD, the pharmacodynamics effects on cellular (ANC, ALC, NLR) and soluble makers (PCT, CRP) of inflammation was investigated. In our previous *post-hoc* study of the CIGMA trial [1] mortality and VFD in the subgroups IgM^low^, CRP^high^, PCT^high^ and IgM^low^ CRP^high^ (not shown). For definitions of subgroups, refer to Table 1 in the main manuscript.

ALC, absolute lymphocyte counts; ANC, absolute neutrophil count; CRP, C-reactive protein, Ig, immunoglobulin; NLR, neutrophil-to-lymphocyte ratio; PCT, procalcitonin; w/o, without.

# *Baseline procalcitonin (PCT) levels in sCAP patients*

| 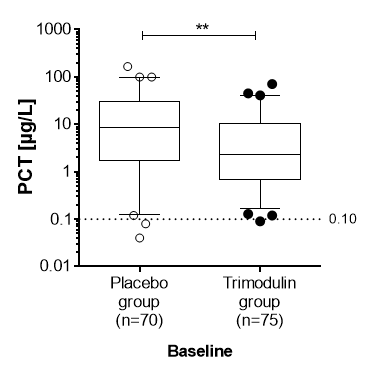 |
| --- |

**Fig. S2: Difference in baseline PCT levels in the placebo and trimodulin groups.**

Box plot showing PCT serum concentration at baseline in patients with sCAP. Line and box represent median (trimodulin: 2.3; placebo: 8.7) with 25–75% percentiles, and whiskers represent 5–95% percentiles. Dots represent single patient baseline values outside the CI. Dotted line represents the normal reference range of PCT (≤0.1 µg/L). **p=0.0016.

CI, confidence interval; PCT, procalcitonin; sCAP, severe community-acquired pneumonia.

# *Immune profile of healthy subjects and of patients with sCAP*

| 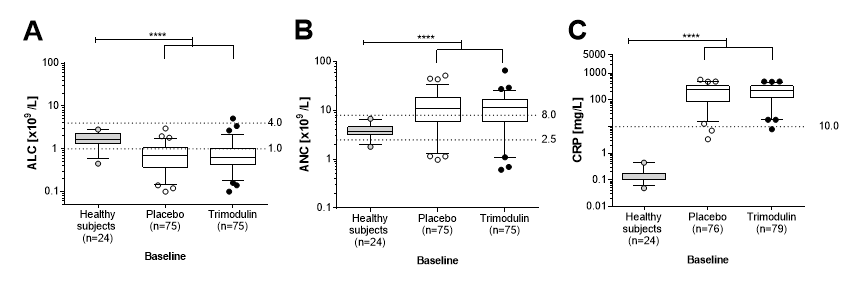 |
| --- |

**Fig. S3: Immune and inflammatory status in healthy subjects and sCAP patients at baseline.**

Box plots showing (**A**) ALC, (**B**) ANC, and (**C**) CRP serum concentration at baseline in healthy subjects (grey, phase I study) and sCAP patients (white, phase II CIGMA trial). Line and box represent median with 25–75% percentiles, and whiskers represent 5–95% percentiles. Dots represent single patient baseline values outside the CI. Dotted lines represent the normal reference ranges.
****p<0.0001 for all indicated comparisons of median values using Mann−Whitney t-test.

ALC, absolute lymphocyte count; ANC, absolute neutrophil count; CI, confidence interval; CRP, C-reactive protein; sCAP, severe community-acquired pneumonia.

# *Impact of trimodulin on ALC in sCAP patients*

| 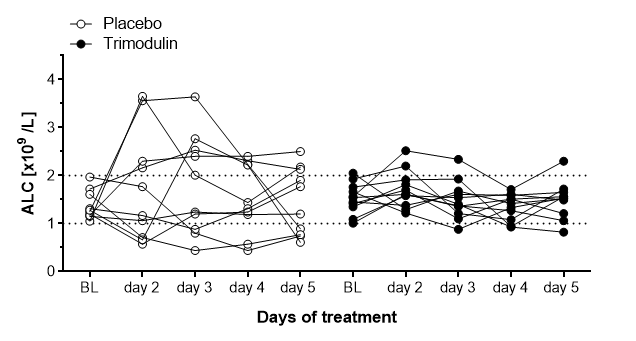 |
| --- |

**Fig. S4: Pharmacodynamic effect of trimodulin on ALC over time.**

Spaghetti plots of ALC in sCAP patients over time. Data included in this time course analysis are from the first 10 patients enrolled in the phase II CIGMA trial who had a baseline ALC of between 1.0 and 2.0 x10^9^/L (dotted lines), in whom a complete data set (up to Day 5) was available and who survived for at least 7 days. Each spaghetti plot represents ALC course over time for a single patient.

ALC, absolute lymphocyte count; BL, baseline; sCAP, severe community-acquired pneumonia.

# *Impact of trimodulin on cellular responses in sCAP patients*

| 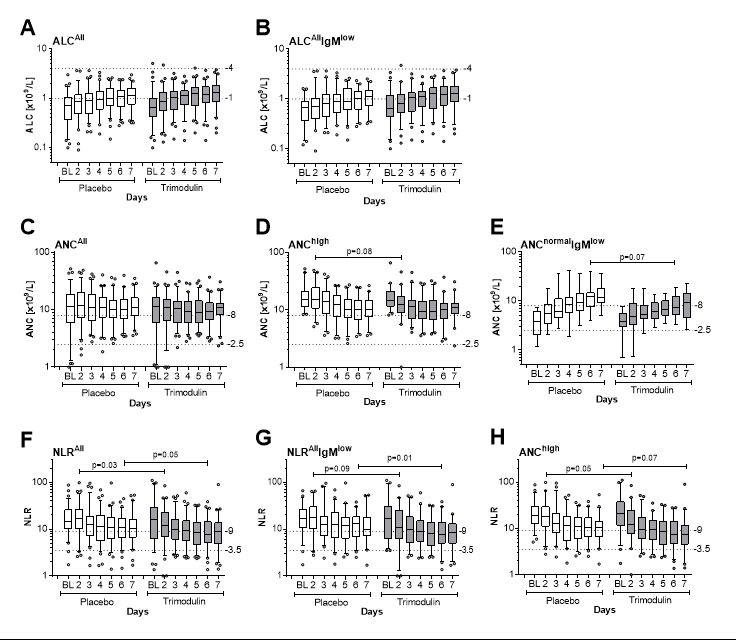 |
| --- |

**Fig. S5: Cellular pharmacodynamic responses modulated by trimodulin in patients with sCAP.**

Box plots of data for ALC (**A**−**B**), ANC (**C**−**E**), and NLR (**F**−**G**) in the different subgroups indicated above each figure. Plots show median (horizontal line), IQR (box), 95% CI (whiskers), and single data points outside the 95% CI (dots). At selected timepoints Mann−Whitney t-tests were performed to determine any statistically significant differences between patients treated with trimodulin or placebo. P-values (significant [p<0.05]; trend [p<0.1]) are indicated above the figures. Please refer to Fig. 2 in the main text for patient numbers and ANOVA results.

ALC, absolute lymphocyte count; ANC, absolute neutrophil count; ANOVA, analysis of variance; BL, baseline; CI, confidence interval; Ig, immunoglobulin; IQR, interquartile range; NLR, neutrophil-to-lymphocyte ratio.

# *Impact of trimodulin on secretion of inflammatory cytokines in vitro*

Trimodulin counteracted the LPS-induced release of pro-inflammatory cytokines by peripheral mononuclear blood cells in a concentration-dependent manner. The investigated cytokines after LPS stimulation included IL-1β, IL-6, and TNF-α (Fig. S6 A–C). Compared with the maximum induction in the absence of trimodulin, cytokine concentrations reduced to ~10% or less after addition of trimodulin at a concentration of 1280 µg/mL. This *in vitro* assay concentration corresponds to 0.29 g/L IgM, assuming 23% IgM in trimodulin. For comparison, in the serum of sCAP patients (n=19, Fig. 1A) the IgM baseline concentrations were between 0.2 and 1.4 g/L. Although these concentrations *in vitro* and in serum cannot be compared directly, they suggest that immune modulation may also occur at physiological IgM concentrations in plasma and that administration of trimodulin would also modulate concentrations in patients.

**Fig. S6: Modulation of pro-inflammatory cytokine responses by trimodulin *in vitro.***

Human peripheral mononuclear cells from healthy donors were stimulated simultaneously at a concentration of 2 x10^5^ cells/mL in RPMI-1640 medium supplemented with 10% FCS with either 1 ng/mL LPS (Donor 3) or 10 ng/mL LPS (Donors 1 and 2) and increasing concentrations of trimodulin (0–1280 µg/mL). After 20 hours incubation, cell supernatant was analyzed using ELISA (R&D systems) for IL-1β (**A**), IL-6 (**B**) and TNF-α (**C**) according to the manufacturer protocols. Two independent experiments were conducted with four replicates each. Dots show mean ±SD.

ELISA, enzyme-linked immunosorbent assay; FCS, fetal calf serum; Ig, immunoglobulin; IL, interleukin; LPS, lipopolysaccharide; RPMI, Roswell Park Memorial Institute; SD, standard deviation; TNF, tumor necrosis factor.

Trimodulin has previously been shown to modulate LPS-induced IL-8 expression, released by neutrophils [2]. Furthermore, similar data from *in vitro* and *in vivo* models of sepsis with the IgM/IgA-enriched preparation Pentaglobin (12% IgM, 12% IgA, 76% IgG) demonstrated a significant reduction in levels of IL-6, TNF-α, and/or IL-1β secreted by monocytes [3–5]. Importantly, in patients with sepsis and septic shock treated with Pentaglobin, IL-10 was also found to decrease [6]. A similar decrease in procalcitonin (PCT) was also shown in patients with sepsis after treatment with Pentaglobin. This was in contrast to control groups in which a second rise in PCT at later timepoints was observed [7–9]. An important aspect to highlight is that this down regulation of PCT and cytokine levels with trimodulin and Pentaglobin persists long-term, which is consistent with a more permanent reduction in systemic inflammation.

Through neutralization, opsonization, and clearance of pathogens and their endotoxins and exotoxins, trimodulin prevents exaggerated toll-like receptor (TLR) activation on the surface of neutrophils and monocytes, preventing the cytokine storm cascade [2, 20, 11]. Furthermore, trimodulin not only modulates cytokine responses by interaction with LPS but may also have a direct impact on the TLR and Fc receptor expression level. Findings from Bohländer et al. and Duerr et al. also support this mechanism [2, 11]. This type of regulation on cytokine release by trimodulin would be expected to have an indirect effect on CRP and PCT. PCT is a marker of infection and inflammation and is upregulated in serum by bacterial and fungal products (pathogen-associated molecular patterns [PAMPs]) and cytokines (IL-1β, IL-2, IL-6, and TNF-α). CRP is a marker of inflammation, an acute phase reactant, and is produced by the liver in response to inflammatory cytokines (IL-1β, IL-6, and TNF-α) and LPS.

# *Impact of trimodulin on soluble markers of inflammation* *in sCAP patients*

| 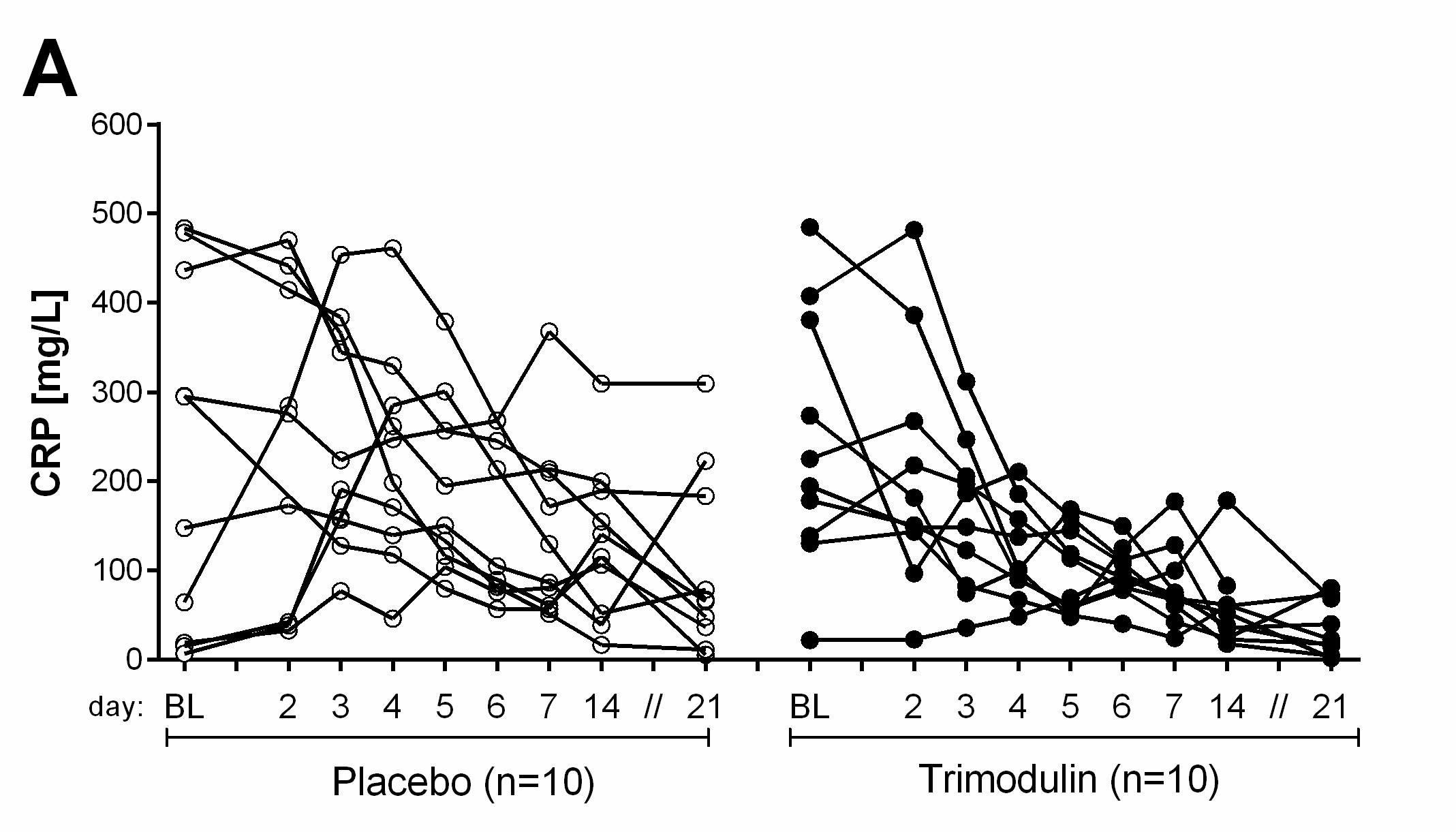 | 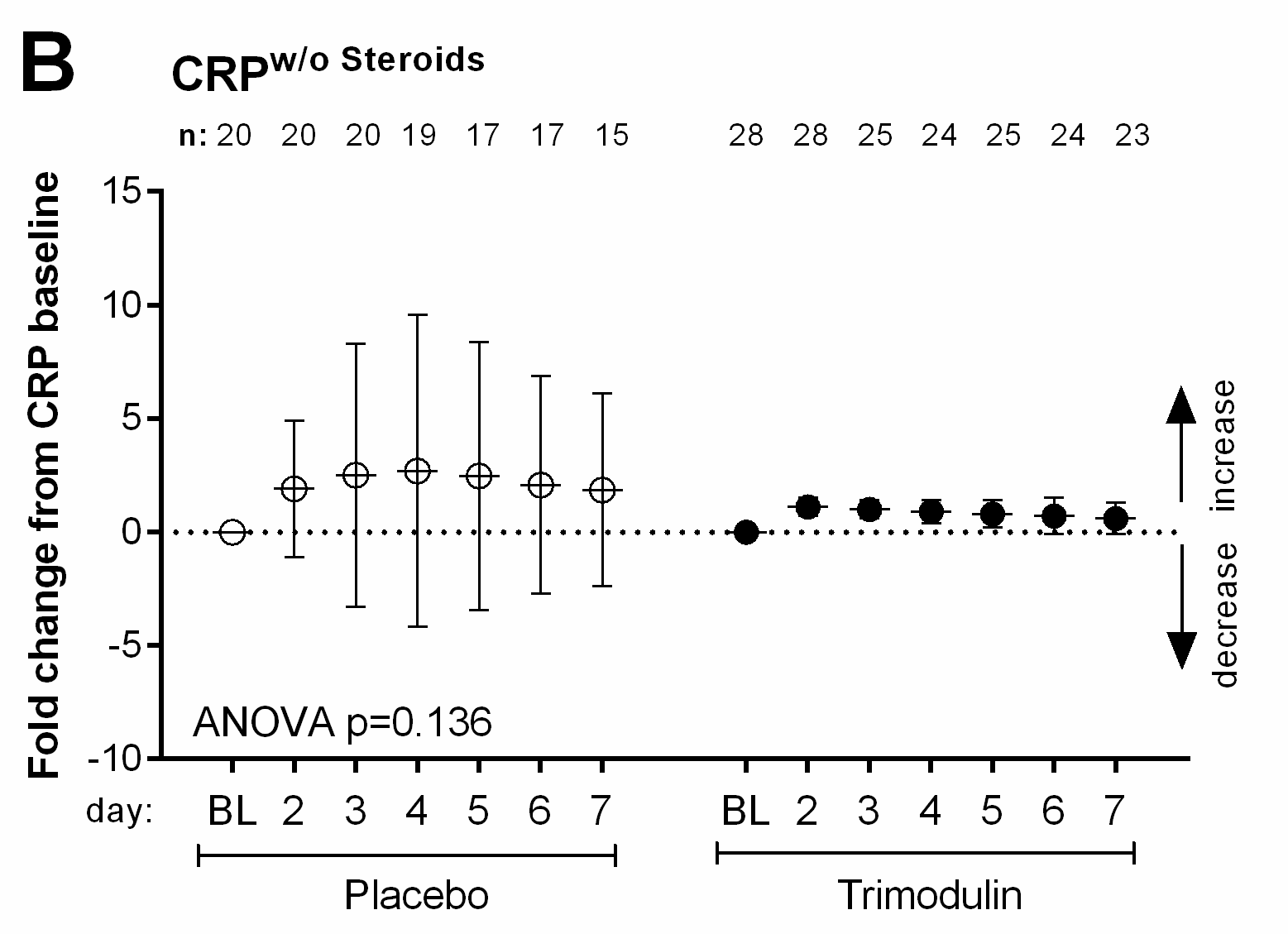 |
| --- | --- |
| 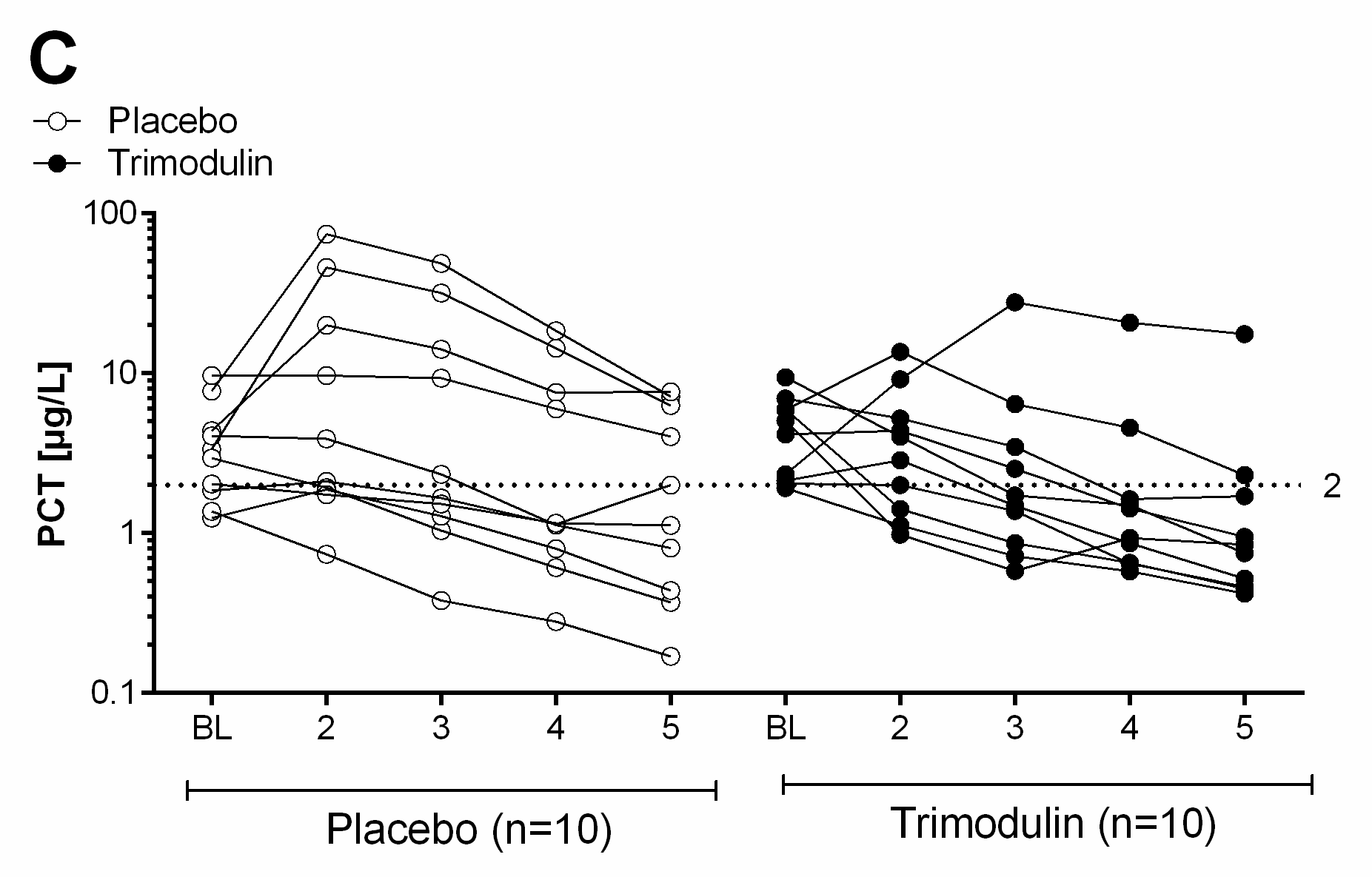 | 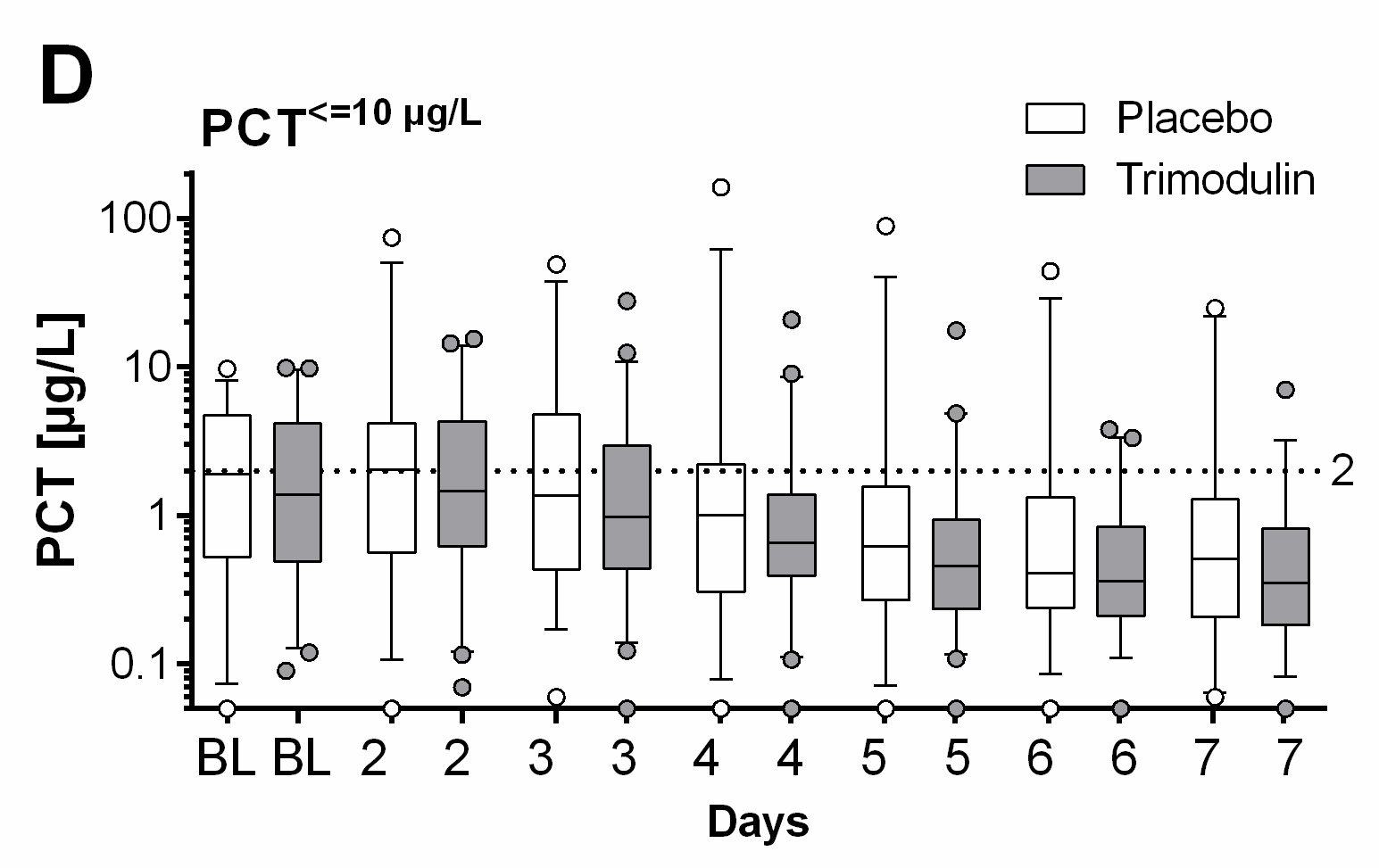 |
| 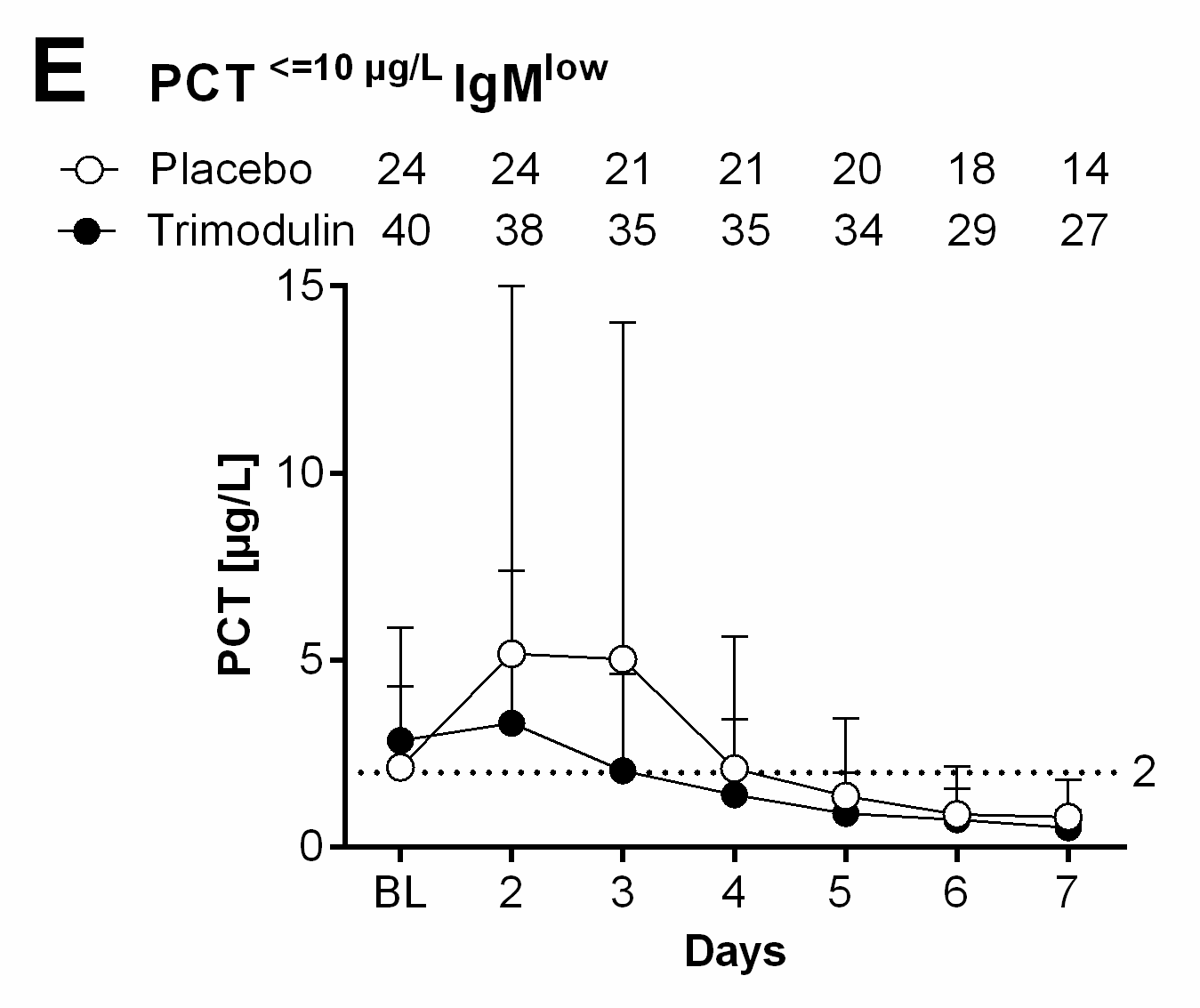 | 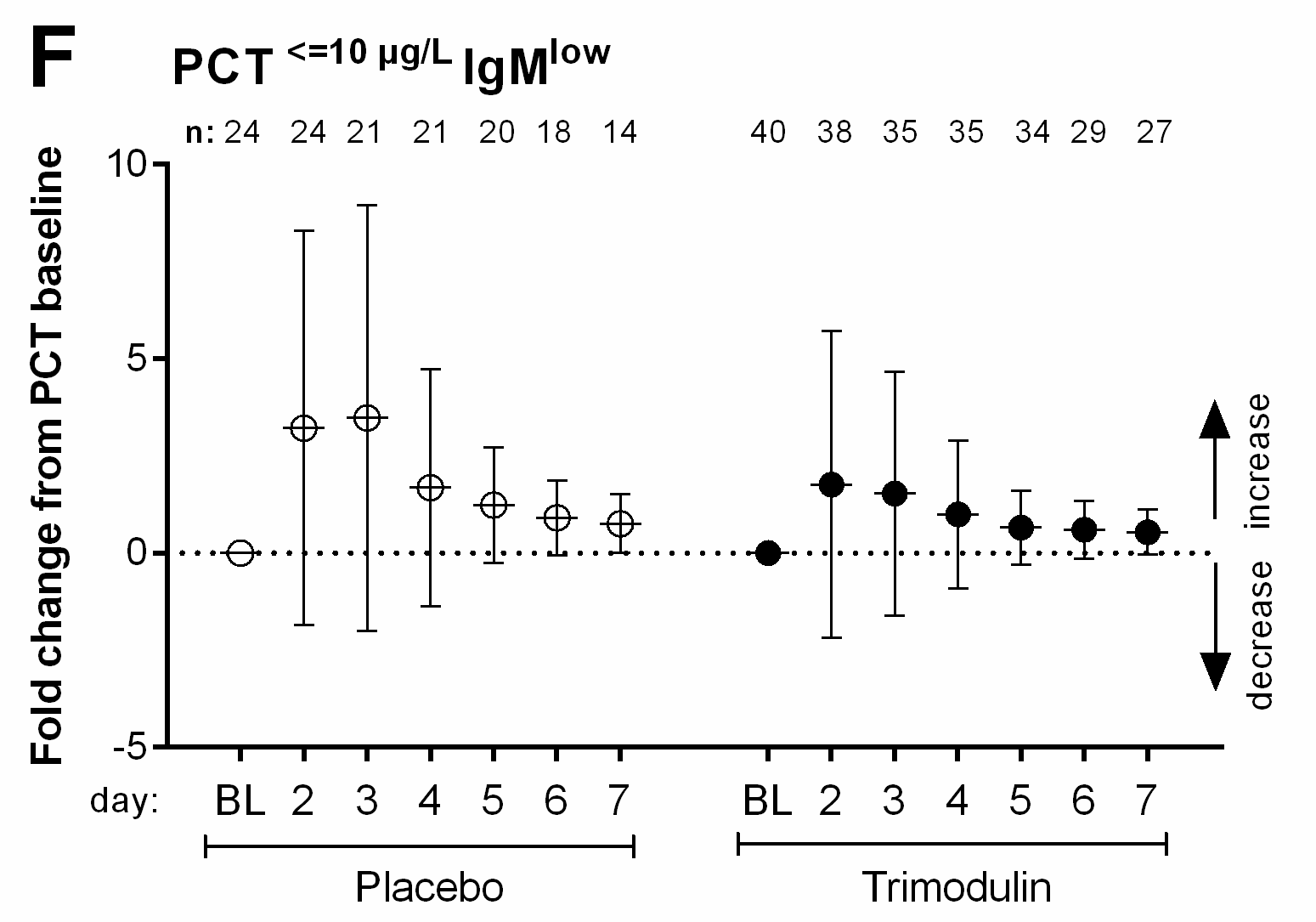 |

**Fig. S7: Modulation of CRP and PCT serum levels by trimodulin.**

**A** Spaghetti plots of CRP concentration–time profiles in sCAP patients. Data included in this time course analysis are from the first 10 patients enrolled in the phase II CIGMA trial in whom a complete data set (up to Day 21) was available. Baseline CRP levels were found to be comparable between the treatment groups. Each spaghetti plot represents CRP course over time for a single patient. **B** Mean fold change from baseline CRP levels in patients who did not receive steroids during the trial (n=48 at baseline). All sCAP patients who received steroids (like methylprednisolone, hydrocortisone, and budesonide) during treatment, regardless of dose, were excluded. Subject numbers are provided above the graph. Repeated measures ANOVA assessed between-subject effects of treatment over time (from baseline to Day 7). **C** Spaghetti plots of PCT values from single sCAP patients (n=10) from baseline through to Day 5 during treatment with placebo or trimodulin. Data included in this time course analysis are from the first 10 patients enrolled in the phase II CIGMA trial who had a baseline PCT of 1−10 µg/L, in whom a complete data set (up to Day 5) was available and who survived for at least 7 days. Each spaghetti plot represents PCT course over time for a single patient. **D−F** is the subgroup of patients (n=64) with PCT ≤10 µg/L and IgM^low^ (≤0.8 g/L) at baseline. **D** Box plot of PCT values in the PCT≤10 µg/L subgroup. Plots show median (horizontal line), IQR (box), 95% CI (whiskers), and single data points outside the 95% CI (dots). **E** Modulation of PCT serum levels by trimodulin in the subgroup of patients with PCT ≤10 µg/L and IgM^low^ (≤0.8 g/L) at baseline. Differences between groups were not statistically significant at any timepoint. **F** Fold change in PCT levels from baseline. The mean ±SD baseline for the trimodulin group was 2.9 ±3.0 µg/L and 2.1 ±2.2 µg/L for placebo group. Differences between groups were not statistically significant at any time point. **C**−**E**: Dotted line: PCT ≥2 µg/L represents a high probability of systemic bacterial infection. **B, F**: The dotted line (0) represents the no-change level at baseline.

ANOVA, analysis of variance; BL, baseline; CI, confidence interval; CRP, C-reactive protein; Ig, immunoglobulin; IQR, interquartile range; PCT, procalcitonin; SD, standard deviation.

# *Impact of indicators of dysregulated inflammatory responses on mortality in placebo-treated sCAP patients*

Low baseline ALC and high ANC were observed in a large proportion of non-surviving sCAP patients in the placebo group (n=18/22, and n=14/22, respectively) (Fig. S8A). More than 90% of the non-surviving patients in the placebo group had both ALC^low^ and ANC^high^ at baseline. Median [IQR] ALC, ANC, and NLR in the non-survivors in the placebo group were 0.62 x10^9^/L [0.35−0.90], 11.5 x10^9^/L [5.1−17.7] and 13.9 [11.9−25.3], respectively.

A large proportion of non-survivors in the placebo group demonstrated high levels of CRP and/or PCT at baseline, above the respective thresholds (n=18/21, and n=16/19, respectively). More than 70% of patients who died in the placebo group (n=13/18) had both high CRP and high PCT at baseline (Fig. S8B). Median [IQR] of CRP and PCT in these non-survivors was 221.2 mg/L [102.0−285.0] and 20.7 µg/L [4.4−59.3], respectively.

A large proportion of non-survivors in the placebo group had low IgM and low IgG levels at baseline (n=17/22 and n=15/22, respectively) (Fig. S8C). Median [IQR] of IgM and IgG in these non-survivors was 0.61 g/L [0.36−0.80] and 6.6 g/L [4.5−9.0], respectively.

Due to the small number of patients, none of the observed differences were significant.

| 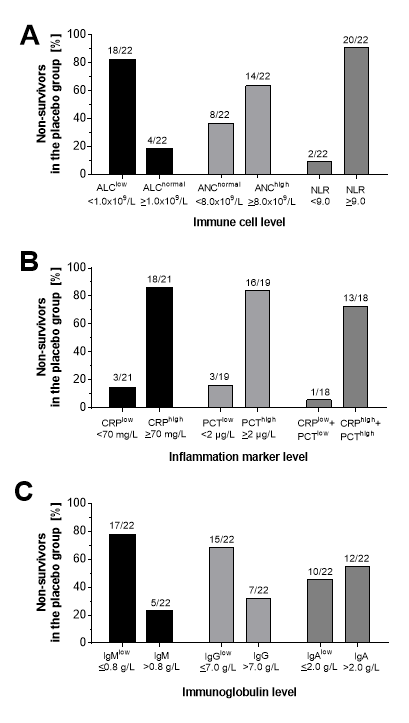 |
| --- |

**Fig. S8: Dysregulated inflammatory responses in non-surviving sCAP patients in the placebo group.**

Bar graph showing the proportions of non-surviving sCAP patients in the placebo group (n=22): **A** With the indicated ALC, ANC, or NLR levels at baseline. **B** With the indicated CRP and PCT levels at baseline (available for n=18−21). Two patients in the placebo group had high CRP and low PCT and two patients *vice versa*. **C** With the indicated Ig levels at baseline.

ALC, absolute lymphocyte; ANC, absolute neutrophil count; CRP, C-reactive protein; Ig, immunoglobulin; NLR, neutrophil-to-lymphocyte ratio; PCT, procalcitonin; sCAP, severe community-acquired pneumonia.

# References

1. Welte T, Dellinger RP, Ebelt H, Ferrer M, Opal SM, Singer M, et al. Efficacy and safety of trimodulin, a novel polyclonal antibody preparation, in patients with severe community-acquired pneumonia: a randomized, placebo-controlled, double-blind, multicenter, phase II trial (CIGMA study). Intensive Care Med. 2018;44:438–48.
2. Bohländer F, Weißmüller S, Riehl D, Gutscher M, Schüttrumpf J, Faust S. The functional role of IgA in the IgM/IgA-enriched immunoglobulin preparation trimodulin. Biomedicines 2021;9(12).
3. Ates I, Dogan N, Aksoy M, Halıcı Z, Gündogdu C, Keles MS. The protective effects of IgM-enriched immunoglobulin and erythropoietin on the lung and small intestine tissues of rats with induced sepsis: Biochemical and histopathological evaluation. Pharmaceutical Biology 2015;53(1):78–84.
4. Vaschetto R, Clemente N, Pagni A, Esposito T, Longhini F, Mercalli F, et al. A double blind randomized experimental study on the use of IgM-enriched polyclonal immunoglobulins in an animal model of pneumonia developing shock. Immunobiology 2017;222(12):1074–80.
5. Barratt-Due A, Sokolov A, Gustavsen A, Hellerud BC, Egge K, Pischke SE, et al. Polyvalent immunoglobulin significantly attenuated the formation of IL-1β in Escherichia coli-induced sepsis in pigs. Immunobiology 2013;218(5):683–9.
6. Domizi R, Adrario E, Damiani E, Scorcella C, Carsetti A, Giaccaglia P, et al. IgM-enriched immunoglobulins (Pentaglobin) may improve the microcirculation in sepsis: a pilot randomized trial. Ann Intensive Care 2019;9(1):135.
7. Meisner M, Tschaikowsky K, Palmaers T, Schmidt J. Comparison of procalcitonin (PCT) and C-reactive protein (CRP) plasma concentrations at different SOFA scores during the course of sepsis and MODS. Crit Care 1999;3(1):45–50.
8. Reith HB, Rauchschwalbe SK, Mittelkötter U, Engemann R, Thiede A, Arnold A, et al. IgM-enriched immunoglobulin (pentaglobin) positively influences the course of post-surgical intra-abdominal infections. Eur J Med Res 2004;9(10):479–84.
9. Tschaikowsky K, Hedwig-Geissing M, Schiele A, Bremer F, Schywalsky M, Schüttler J. Coincidence of pro- and anti-inflammatory responses in the early phase of severe sepsis: Longitudinal study of mononuclear histocompatibility leukocyte antigen-DR expression, procalcitonin, C-reactive protein, and changes in T-cell subsets in septic and postoperative patients. Crit Care Med 2002;30(5):1015–23.
10. Bohländer F, Riehl D, Weißmüller S, Gutscher M, Schüttrumpf J, Faust S. Immunomodulation: immunoglobulin preparations suppress hyperinflammation in a COVID-19 model via FcγRIIA and FcαRI. Front Immunol 2021;12:700429.
11. Duerr C, Bacher A, de Martin A, Sachet M, Sadeghi K, Baumann S, et al. The novel polyclonal Ab preparation trimodulin attenuates *ex vivo* endotoxin-induced immune reactions in early hyperinflammation. Innate Immun 2019;25(6):374–88.
